# Cloned, CD117 Selected Human Amniotic Fluid Stem Cells Are Capable of Modulating the Immune Response

**DOI:** 10.1371/journal.pone.0026535

**Published:** 2011-10-26

**Authors:** Emily C. Moorefield, Elizabeth E. McKee, Luis Solchaga, Guisseppe Orlando, James J. Yoo, Steve Walker, Mark E. Furth, Colin E. Bishop

**Affiliations:** 1 Wake Forest Institute for Regenerative Medicine, Winston-Salem, North Carolina, United States of America; 2 Center for Stem Cell and Regenerative Medicine, Case Western Reserve University, Cleveland, Ohio, United States of America; 3 Transplantation Research Immunology Group, Nuffield Department of Surgical Sciences, University of Oxford, Oxford, United Kingdom; University of Medicine and Dentistry of New Jersey, United States of America

## Abstract

Amniotic fluid stem (AFS) cells are broadly multipotent, can be expanded extensively in culture, are not tumorigenic and can be readily cryopreserved for cell banking. Mesenchymal stem cells (MSC) show immunomodulatory activity and secrete a wide spectrum of cytokines and chemokines that suppress inflammatory responses, block mixed lymphocyte reactions (MLR) and other immune reactions, and have proven therapeutic against conditions such as graft-versus-host disease. AFS cells resemble MSCs in many respects including surface marker expression and differentiation potential. We therefore hypothesized that AFS cells may exhibit similar immunomodulatory capabilities. We present data to demonstrate that direct contact with AFS cells inhibits lymphocyte activation. In addition, we show that cell-free supernatants derived from AFS cells primed with total blood monocytes or IL-1β, a cytokine released by monocytes and essential in mediation of the inflammatory response, also inhibited lymphocyte activation. Further investigation of AFS cell-free supernatants by protein array revealed secretion of multiple factors in common with MSCs that are known to be involved in immune regulation including growth related oncogene (GRO) and monocyte chemotactic protein (MCP) family members as well as interleukin-6 (IL-6). AFS cells activated by PBMCs released several additional cytokines as compared to BM-MSCs, including macrophage inflammatory protein-3α (MIP-3α), MIP-1α and Activin. AFS cells also released higher levels of MCP-1 and lower levels of MCP-2 compared to BM-MSCs in response to IL-1β activation. This suggests that there may be some AFS-specific mechanisms of inhibition of lymphocyte activation. Our results indicate that AFS cells are able to suppress inflammatory responses in vitro and that soluble factors are an essential component in the communication between lymphocytes and AFS cells. Their extensive self-renewal capacity, possibility for banking and absence of tumorigenicity may make AFS cells a superior source of stable, well characterized “off the shelf” immunomodulatory cells for a variety of immunotherapies.

## Introduction

Tissue engineering and cell therapy will be enhanced by improved cell sources. Mesenchymal stromal cells (MSCs), an adherent population found in nearly every adult tissue but most often obtained from bone marrow (BM-MSCs) or adipose tissue, have been examined for multiple clinical purposes [1], [2], [3], [4], [5]. MSCs can give rise to differentiated cells of the mesodermal lineage including bone, fat, cartilage, tendon and muscle [6], [7], [8]. In addition, their ability to evade immunosurveillance after cell transplantation and to suppress the immune response has made BM-MSCs a particularly attractive candidate for clinical use [9], [10]. In particular, it was observed that BM-MSCs could suppress lymphocyte proliferation and activation in response to allogeneic activation or chemical stimulation *in vitro* or *in vivo* [8], [11], [12].

Immunoregulation by BM-MSCs is thought to result from both direct interactions between the stromal and immune cells [13], [14], [15] and the actions of anti-inflammatory soluble factors released by the stromal cells [2], [16]. The secretion of these factors occurs in response to pro-inflammatory signals from the local environment, including IFN-γ, TNF-α, IL-1α and IL-1β [17], [18], [19]. Clinical applications for which the trophic action of BM-MSCs may prove valuable include support of hematopoietic transplantation and the treatment of graft versus host disease (GvHD), osteogenesis imperfect, and acute myocardial infarction [20], [21], [22], [23], [24]. However, the relatively limited proliferation of BM-MSCs under standard conditions suitable for manufacture of a clinical product presents a potential drawback for their medical application [25]. For this reason, we sought to determine whether amniotic fluid-derived stem (AFS) cells, which display considerably greater expansion capacity and appear well suited to large-scale banking [26] possess comparable immunomodulatory capability.

The amniotic fluid contains multiple cell types derived mainly from exfoliating surfaces of the developing fetus [27]. These include cells from the fetal skin, respiratory system, urinary and gastrointestinal tracts, along with populations of MSCs [28]. De Coppi et al. (2007) described a novel population of multipotent stem cells from amniotic fluid obtained by immunoselection for c-Kit (CD117), the cell surface receptor for stem cell factor (SCF), and designated them amniotic fluid-derived stem (AFS) cells. AFS cells are characterized by their high capacity for self-renewal and their ability to differentiate to toward lineages representative of all three germ layers including hepatocytes, osteocytes, chondrocytes and adipocytes [26], [29]. Some clonal AFS cell lines were shown to proliferate *in vitro* well past Hayflick's limit (greater than 80 population doublings) with no signs of malignant transformation, chromosomal abnormalities, or loss of differentiation potential [26]. AFS cells and BM-MSCs share many characteristics including expression of the surface markers CD29, CD44, CD73, CD90 and CD105. However, AFS cells also express the more primitive stem cell marker SSEA4 [26]. The two cell types also share a similar immune antigen surface profile with positive MHC Class I expression but little to no MHC Class II expression. MSCs also have the ability to avoid allogeneic rejection [30]. We hypothesized that cells in the amniotic fluid may have immunopriviledged status, as fetal cells must possess mechanisms to avoid destruction by the maternal immune system during development [31]. We further hypothesized that, like MSCs, AFS cells also possess immunosuppressive properties. There has been a single report that unselected mesenchymal stromal cells from amniotic fluid inhibit lymphocyte proliferation *in vitro* [32]. We sought to determine whether cloned lines of CD117-selected AFS cells could modulate immune responses.

## Materials and Methods

### AFS Cell and MSC Isolation and Culture

Human AFS cells were isolated as previously reported [26]. Briefly, back-up cultures from human amniocentesis were obtained from the clinical cytogenetics laboratory and expanded on petri dishes in α-MEM medium (Invitrogen, Carlsbad, CA) containing 15% ES-FBS, 1% glutamine and 1% penicillin/streptomycin (Invitrogen, Carlsbad, CA) and supplemented with 18% Chang B and 2% Chang C (Irvine Scientific, Irvine, CA). Cultures were maintained at 37 C in a 5% CO_2_ atmosphere. Once confluent, cells were immunoselected based on CD117 expression. Cultures were trypsinized and single-cell suspensions labeled with a rabbit polyclonal antibody against CD117 (Santa Cruz Biotechnology, Santa Cruz, CA), then labeled with Goat Anti-Rabbit IgG MicroBeads and selected on a Mini-MACS apparatus (Miltenyi Biotech, Auburn, CA) following the protocol recommended by the manufacturer. AFS cell lines A1 and H1 between passages 15 and 20 were used for these experiments [26].

Human BM-MSCs were derived and cultured as previously reported [33]. BM-MSC isolates were kindly provided by L. Solchaga and cells between passages 3 and 5 were used for these experiments.

### Immunophenotype analysis of AFS cells and BM-MSCs

Human AFS cells and BM-MSCs at 70% confluence were trypsinized and resuspended in PBS with 1% FBS. Cells were incubated with FcR Block (Miltenyi Biotech, Auburn, CA) at room temperature for 5 minutes, and then incubated with saturating amounts of antibodies on ice for 30 minutes. The following FITC- or PE- conjugated antibodies recognizing human antigens were used: CD80, CD86, HLA-DR, HLA-ABC (BD Biosciences, San Diego, CA). Each analysis included corresponding FITC- and/or PE- conjugated isotype controls. Samples were run on a Becton Dickinson FAC-Scan flow cytometric system (BD Biosciences, San Diego, CA) and analysis was completed using FlowJo Software (FlowJo, Ashland, OR).

### Interferon-gamma ELISPOT analysis (Phytohaemagglutinin (PHA) activation assay)

Elispot analysis was performed as described previously by Maitra et al. (2006). Briefly, Multiscreen filter 96-well plates for Elispot (Millipore, Billerica, MA) were coated with human interferon-γ (IFN-γ) capture antibody (2G1; Endogen, Rockford, IL). Peripheral blood mononuclear cells (PBMCs) were acquired from AllCells (Emeryville, CA), activated with 5 µg/ml PHA (L1668; Sigma, St. Louis, MO) and cultured in the IFN-γ coated wells with increasing stem cell densities or stem cell conditioned medium. Experiments examining cell contact included wells with 150,000 PBMCs, 5 µg/ml PHA (L1668; Sigma, St. Louis, MO) and increasing amounts of AFS cells or BM-MSCs (“stem cells”) ranging from 4,688 (1∶32 stem cell to PBMC ratio) to 75,000 stem cells (1∶2 stem cell to PBMC ratio). Controls included unstimulated PBMCs and stimulated PBMCs with no stem cells. A minimum of quadruplicate wells for each condition was analyzed in at least 3 independent experiments and a single representative experiment is shown. After 24 hours, plates were washed and incubated with biotinylated detection antibody (B133.5; Endogen, Rockford, IL) for 2 hours at 37 C. Streptavidin-Horse Radish Peroxidase (HRP) (P0397; Dako, Carpinteria, CA) was then added and incubated for 1 hour at room temperature. IFN-γ spots were visualized after incubation with 3-amino-9-ethylcarbazole (34004; Pierce, Rockford, IL). An Elispot image analyzer was used to quantify the number of spots in each well (Immunospot Cellular Technology, Shaker Heights, OH). The percent activation for a given condition was derived as the ratio of the mean spot number to the mean spot number of the positive control, multiplied by 100.

### Transwell experiments

Monolayers of AFS cells or BM-MSC were cultured in the presence of total blood monocytes separated by trans-well chambers (Corning, Corning, NY) or in the presence of IL-1β. In experiments examining the effect of conditioned medium on the immune assay each well included 150,000 PBMCs, 5 µg/ml PHA and 24 hour conditioned medium from one of the following sources: 1) Basal medium, 2) total blood moncytes, 3) AFS cells, 4) AFS cells cultured with total blood monocytes, 5) AFS cells cultured with IL-1β, 6) BM-MSCs, 7) BM-MSCs cultured with total blood monocytess, 8) BM-MSCs cultured with IL-1β. Controls included unstimulated PBMCs, which produced no IFN-γ, and stimulated PBMCs in growth medium, which produced the maximum amount of IFN-γ. A minimum of quadruplicate wells for each condition was analyzed in at least 3 independent experiments and a single representative experiment is shown. The Elispot plate was developed and analyzed as described previously.

### Cytokine secretion by antibody array

Cell-free supernatants from cells cultured under the eight conditions outlined above were analyzed for cytokine secretion using Cytokine Antibody Array V, VI, VII (RayBiotech, Norcross, GA). Antibodies against each of 174 cytokines, chemokines and growth factors were spotted onto the array membrane in duplicate. After blocking for 30 minutes, the membranes were incubated with 200 µl of supernatant at room temperature for 2 hours. Then primary biotin-conjugated antibody was added to each well and incubated at room temperature for 2 hours. Horseradish peroxidase-conjugated streptavidin was then added to each well and incubated at room temperature for 30 minutes. The wells were developed by addition of detection buffer and analyzed using a luminescent image analyzer system (GenePix4000B, Axon Instruments, Union City, CA).

### Semi-Quantification and analysis of cytokine array data

Semi-quantification of cytokine levels was achieved using Axon software. The pixel density of each spot was measured and background levels from negative controls were subtracted. The intensity of positive control spots was used to normalize results between the three membranes. The intensity for each cytokine was then averaged over the duplicate spots. Controls of serum containing medium, total blood monocytes cultured alone or stem cells cultured alone were included in the array and these values were subtracted as background from the appropriate samples.

### Statistics

Results are expressed as mean +/− standard deviation. The statistical significance was determined by one way analysis of variance (ANOVA). A p value<0.05 was considered statistically significant.

## Results

The purpose of these experiments is to compare stem cells derived from amniotic fluid to mesenchymal stromal cells derived from bone marrow in their ability to dampen the immune response *in vitro*.

### Immunogenic characterization of amniotic fluid stem cells

**Figure 1:**
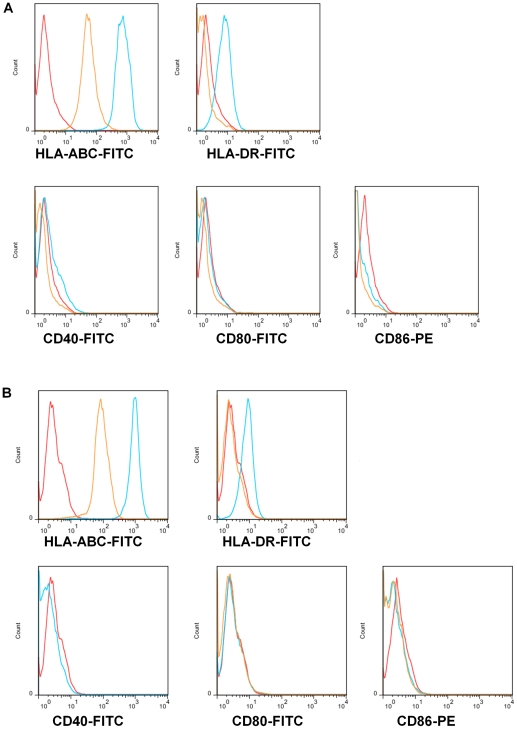
The effect of IFN-γ on the immunophenotype of AFS cells and BM-MSCs. BM-MSCs (A) or AFS cells (B) were cultured under growth conditions with (blue) and without (yellow) addition of IFN-γstimulation for 24 hours. The cells were incubated with fluorescently labeled antibodies against the the major histocompatibility complex molecules MHC-I and MHC-II as well as the costimulatory molecules CD40, CD80 and CD86. The red line shows appropriate isotype controls. A. BM-MSCs under growth conditions express high levels of MHC-I and no MHC-II, CD40, CD80 or CD86. Upon IFN-γ stimulation MSCs upregulate MHC-I and MHC-II while CD40, CD80 and CD86 remain unchanged. B. AFS cells show similar expression patterns to MSCs with high levels of MHC-I and no MHC-II, CD40, CD80 or CD86. AFS cells also upregulate MHC-I and MHC-II in response to IFN-γ stimulation. Before stimulation they express high levels of MHC-I but low levels of MHC-II, CD40, CD80 and CD86. After IFN-γ stimulation the levels of MHC-I and MHC-II expression increase but CD80 and CD86 expression remains unchanged.

We carried out flow cytometry to directly compare the expression of immune-related surface markers between human AFS cells and bone marrow-derived MSCs (BM-MSCs) (Figure 1). Consistent with previous reports, the BM-MSCs were positive for major histocompatibility complex (MHC) – Class I but expressed no detectable MHCClass II (Figure 1A). The BM-MSCs also showed no expression of the costimulatory molecules CD-80 and CD86. AFS cells likewise demonstrated positive expression of MHC – Class I and undetectable expression of MHC – Class II, CD-80 and CD-86 (Figure 1B). To compare their response to IFN-γ, AFS cells and BM-MSCs were cultured in the presence of the cytokine for 24 hours and flow cytometry was performed to examine the expression of the same immune response related surface markers. We found that IFN-γ induced up-regulation of MHC I and II, but not CD40, CD80, or CD86, in both BM-MSCs (Figure 1A) and AFS cells (Figure 1B).

### AFS cells suppress lymphocyte activation

**Figure 2:**
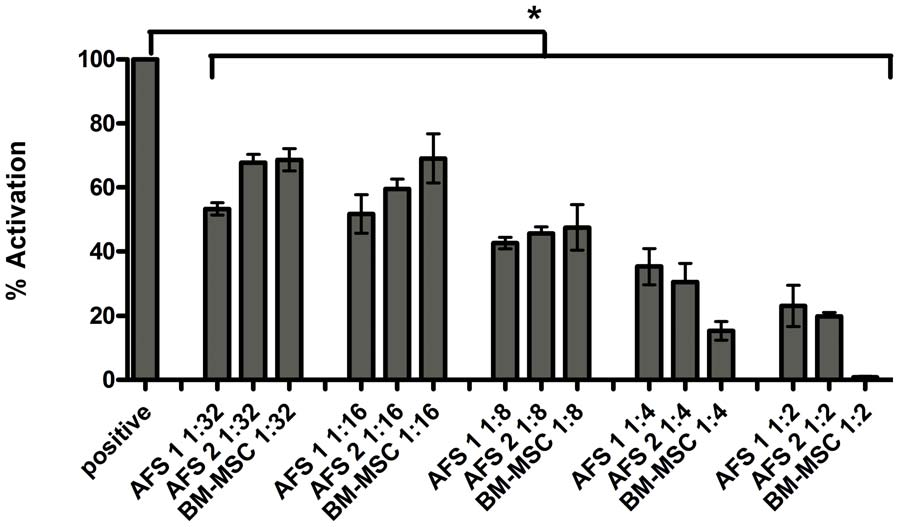
Human AFS cells inhibit lymphocyte activation in a dose dependent manner similar to that of BM-MSCs. Immunoassays assessing lymphocyte activation were performed on two independent amniotic fluid stem cell lines (AFS1 and AFS2) or bone marrow-mesenchymal stem cell (BM-MSC) isolates. T lymphocytes were activated with phytohemaaglutinin (PHA) and cultured in 96 well plates coated with IFN-γ capture antibody in the presence of increasing amounts of stem cells from 1∶32 (4,688 stem cells cultured with 150,000 PBMCs) to 1∶2 (75,000 stem cells cultured with 150,000 PBMCs) for 24 hours. Positive control wells contained lymphocytes activated with PHA and negative control wells included unactivated lymphocytes. Lymphocyte activation was assessed by counting the number of lymphocyte clones producing IFN-γ. Activation is expressed as a percentage of the positive control wells. Both AFS lines and BM-MSC inhibited T-cell compared to the PHA activated control to an approximately equal extent, and was dependant on the number of stem cells added. Inhibition varied from about 40% at a 1∶32 ratio to 80–90% inhibition at the highest ratio of 1∶2.

The ability of AFS cells to inhibit lymphocyte activation by PHA was examined in an Elispot assay measuring the pro-inflammatory cytokine IFN-γ. Two independent AFS cell lines, a BM-MSC isolate and a human dermal fibroblast were tested in this assay. Both AFS cell lines and the BM-MSC isolate induced exerted dose-dependent inhibition of lymphocyte activation (Figure 2). At low numbers of stem cells, the extent of inhibition of lymphocyte activation by the AFS cells was essentially equivalent to that for BM-MSCs. At a ratio of 1 stem cell, either AFS or BM-MSC, to 32 lymphocytes, the percent activation was about 70 percent of the positive control wells which contained no stem cells. When the stem cell number was increased two-fold, lymphocyte activation was further inhibited to 60 percent. By increasing the number of stem cells again by two-fold we showed decreased activation of lymphocytes to about 50 percent of the positive control values. At the highest levels of stem cells, BM-MSCs appeared slightly more potent than AFS cells, and completely inhibited PHA-stimulated activation at the highest dose tested. By contrast, control dermal fibroblasts did not inhibit lymphocyte activation at any concentration.

### Conditioned medium from AFS cells suppresses lymphocyte activation

**Figure 3:**
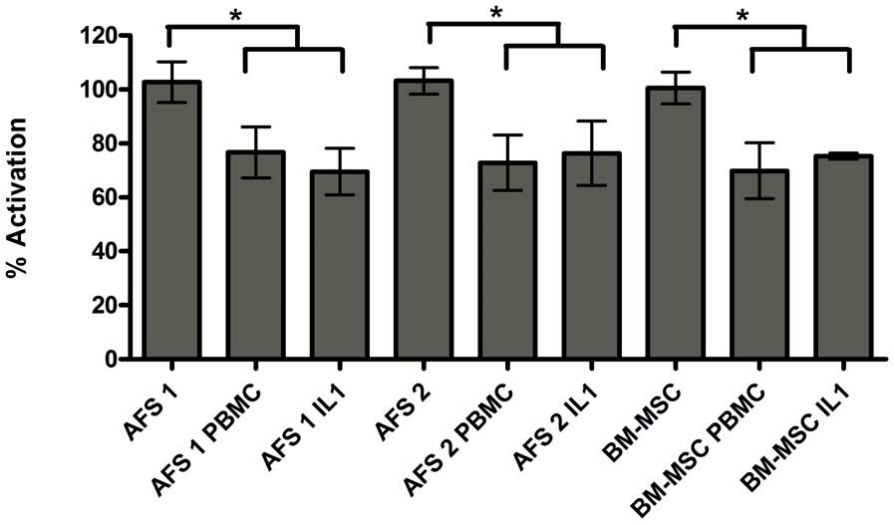
AFS mediated immunosuppression does not require cell-cell contact. Amniotic fluid stem (AFS) cells from two independent sources or bone marrow derived mesenchymal stem cells (BM-MSCs) were cultured under growth conditions (AFS 1, AFS 2, BM-MSC) or activated by co-culture with total blood monocytes (AFS 1 PBMC, AFS 2 PBMC, BM-MSC PBMC) or IL-1β (AFS 1 IL1, AFS 2 IL1, BM-MSC IL1) to release soluble factors. One way mixed lymphocyte reactions (MLR) were incubated in the presence of 24 hour conditioned medium from stem cells cultured with either peripheral blood mononuclear cells (PBMCs) or IL-1β. PHA activated lymphocytes were cultured in 96 well plates coated with IFN-γ capture antibody in the presence of conditioned mediums for 24 hours. Positive control wells contained lymphocytes activated with PHA and negative control wells contained unactivated lymphocytes. Lymphocyte activation was assessed by counting the number of clones producing IFN-γ. Percent activation was calculated by comparing wells containing stem cells to positive control wells. It can be seen that all supernatants were capable of inhibiting T-cell activation by PHA by approximately 20–25%. All conditions are statistically significant when compared to the positive control wells.

To examine the possibility that AFS cells secrete immunosuppressive factors, we assessed the effect of conditioned medium (CM) in the lymphocyte activation assay. CM was prepared from AFS cells and BM-MSCs with and without pre-treatment with the pro-inflammatory cytokine IL-1β. CM also was prepared from mixed cultures of AFS cells or BM-MSCs with PBMCs. CM from unstimulated AFS cells or BM-MSC showed minimal suppression (less than 10% reduction) of lymphocyte activation (Figure 3). CM from AFS cells or BM-MSCs stimulated with IL-1β both showed significantly enhanced suppression of PBMC activation, to 60–70 percent of control levels (Figure 3). CM obtained after stimulation of AFS cells or BM-MSCs by co-culture with PBMCs also significantly suppressed lymphocyte activation to a similar extent compared to CM from IL-1β-stimulated cells.

### Identification of secreted cytokines and growth factors

**Figure 4:**
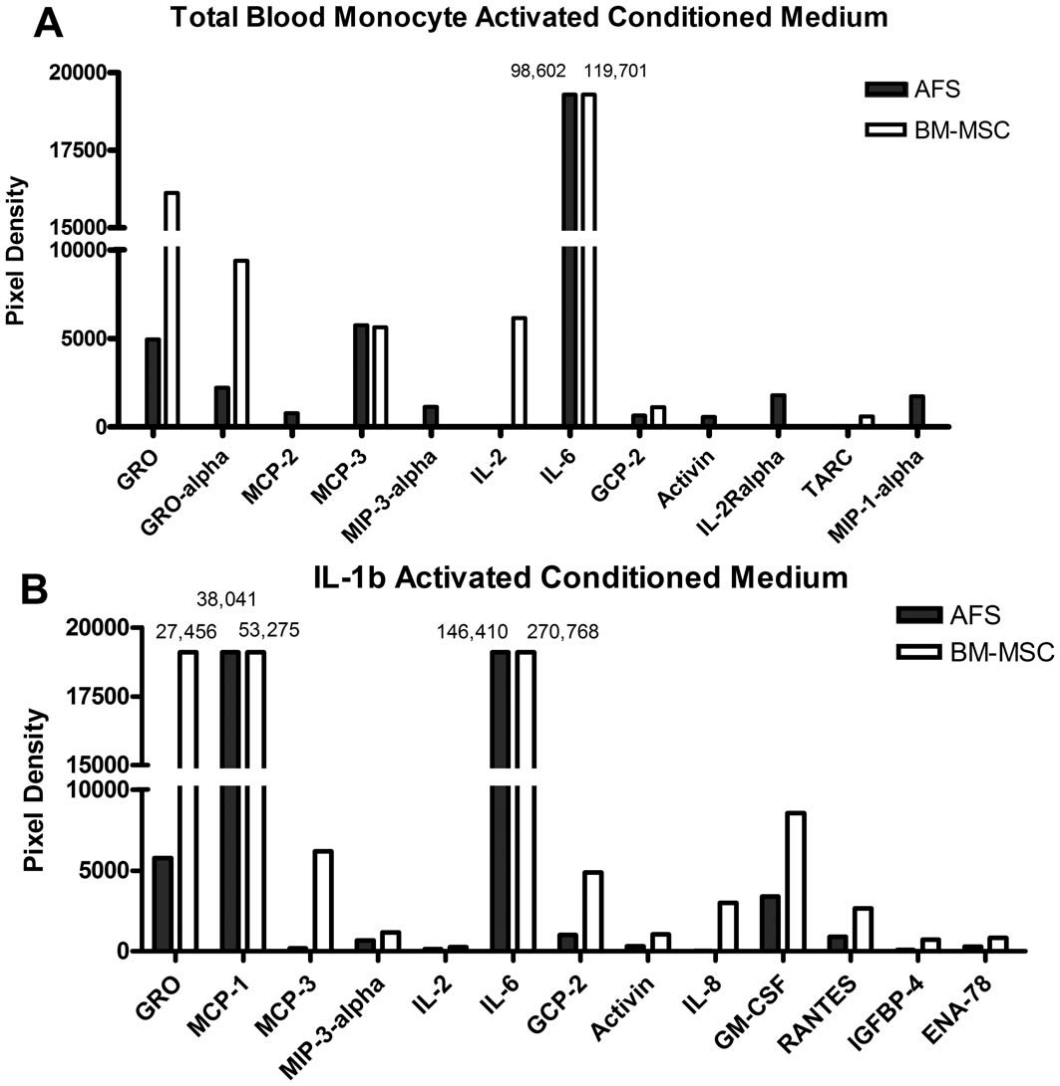
Soluble factors released from AFS cells and BM-MSCs in response to activation. Amniotic fluid stem (AFS) cells or bone marrow derived mesenchymal stem cells (BM-MSCs) were activated by culture with A. total blood monocytes or B. IL-1β and the cytokines released were measured by cytokine array. Background cytokine levels were subtracted to normalize samples and include cytokines released by PBMCs cultured alone and stem cells cultured alone. Quantification of protein optical density was measured using GenePix4000 software.

Medium conditioned by activated AFS cells and BM-MSCs were examined by protein array to determine factors important in immune response regulation. We assessed levels of 274 different cytokines, chemokines and growth factors in CM from AFS cells and BM-MSCs cultured under standard growth conditions or activated with either PBMCs (Figure 4A) or IL-1β (Figure 4B). Controls to assess background levels of soluble factors included serum containing medium and PBMCs cultured alone. Two identical arrays were completed on different days, one including conditioned medium from 1) Cell free medium, 2) AFS cells, 3) AFS cells activated with PBMCs and 4) AFS cells activated with IL-1β and the other including 1) PBMC conditioned medium, 2) BM-MSCs, 3) BM-MSCs activated with PBMCs, 4) BM-MSCs activated with IL-1β.

Amniotic fluid stem cells and BM-MSCs under growth conditions released low levels of very few cytokines. However, upon activation by either PBMCs or IL-1β elevated levels of cytokines were released and a similar cytokine profile was seen from both AFS cells and BM-MSCs. Activation by PBMCs caused release of high levels of several cytokines by AFS cells and BM-MSCs including growth related cytokine – α (GRO-α), monocyte chemotactic protein-3 (MCP-3) and interleukin – 6 (IL-6). Granulocyte chemotactic protein -2 (GCP-2) and interleukin – 2 (IL-2) receptor alpha (IL-2Rα) were also released by both cell types, although to a lesser extent. While PBMC activated AFS cells released monocyte chemotactic protein-2 (MCP-2), macrophage inflammatory protein 1α (MIP-1α), MIP-3α and Activin while BM-MSCs did not.

Culture with pro-inflammatory cytokine IL-1β resulted in a cytokine release profile similar to that seen with PBMC activation. Both AFS cells and BM-MSCs released detectable levels of GRO, GRO-α, MCP-1, IL-2, IL-6 and granulocyte macrophage colony stimulating factor (GM-CSF). Each stem cell type also released low levels of the cytokines MIP-3α, IL-2, regulated upon activation, normal T-cell expressed, and secreted (RANTES) and epithelial neutrophil-activating protein-78 (ENA-78). BM-MSCs released detectable levels of several additional cytokines upon IL-1β stimulation including GCP-2, Activin, IL-8 and insulin-like growth factor-binding protein 4 (IGF-BP4).

## Discussion

Human amniotic fluid stem (AFS) cells are broadly multipotent, derived from an immunoprivileged site, can be expanded extensively in culture, and do not form tumors when implanted in immune deficient mice. We assessed AFS cells for immunomodulatory properties, compared to bone marrow-derived mesenchymal stromal cells (BM-MSCs). Characterization of AFS cell surface markers revealed high expression of major histocompatibility (MHC) Class I and lack of MHC Class II or co-stimulatory molecules (CD80, CD86, CD40) consistent with previous reports [8], [16], [26]. Interferon-gamma (IFN-γ) greatly increased expression of both MHC Class I and MHC Class II. This indicates that AFS cells, like MSCs, may not strongly activate rejection responses in allogeneic hosts. We further observed that AFS cells, again like MSCs, inhibit phytohemagglutinin (PHA)-induced activation of lymphocytes in a dose-dependent manner. The mechanism of immunosuppression by AFS cells involves both direct cell-cell contact between the stem cells and the immune cells and indirect interaction through immunosuppressive factors secreted by the stem cells in response to activation. Analysis of cell-free supernatants demonstrated a substantial overlap in the cytokine release profiles of AFS cells and BM-MSCs, either at rest or when activated with PBMCs or interleukin 1-beta (IL-1β). In response to activation, the most highly upregulated protein in both cell types was interleukin 6 (IL-6). IL-6 is a broad-acting cytokine involved in the control of the immune response as well as stem cell development and regulation [20], [34]. Mesenchymal stem cells derived from cord blood (CB-MSCs) and BM-MSCs have also been reported to secrete high amounts of IL-6 when activated with IL-1β [35]. The biological relevance of this IL-6 response likely lies in both local and systemic protection against inflammation [36], [37]. BM-MSCs and AFS cells also released high levels of growth related oncogene family members GRO and GRO-α as well as monocyte chemotactic protein-1 (MCP-1) upon IL-1β activation. These chemokines have well known effects on cells of the immune system and are important in inflammation and wound healing. GRO acts on neutrophils and MCP-1 acts mainly on macrophages, recruiting them to sites of inflammation [38].

Compared to BM-MSCs, activated AFS cells released significantly higher levels of several cytokines including MCP-2, MIP-3α and MIP-1α whereas the activated BM-MSCs released significantly higher levels of GRO-α, MCP-3, GCP-2 and IL-8. These data suggest that AFS cells may possess some alternative molecular mechanisms to modulate the immune response. In this regard, we have recently compared the effect of rat AFS (rAFS) and rat BM-MSC (rBM-MSC) in a rat model of experimental necrotising enterocolitis (NEC). We were able to show that rAFS cells significantly improved the survival and enhanced the repair of the damaged intestine, whereas rBM-MSC had no effect (Paolo De Coppi, personal communication). Similar results have been achieved using human AFS cells to repair the kidney and restore its function in an immunodeficient mouse model of acute tubular necrosis (ATN) [39]. In this model AFS cells appear to possess immunomodulatory function, initiating the release of several murine anti-inflammatory cytokines and reducing the release of pro-inflammatory cytokines [39].

Current stem cell therapies using MSCs require large cell doses and as a result of the limited growth potential of MSCs, cells from several donors must be pooled for a single treatment, a practice which results in variation between treatments. AFS cells have the unique advantage of extensive self-renewal *in vitro* allowing for long-term expansion, full characterization and cryopreservation. These factors make AFS cells an ideal source for cell banking and standardized ‘off the shelf’ therapeutic use, allowing them to serve as a reproducible immunomodulatory cell therapy source especially for autoimmune-type diseases.
